# Extreme temperature events reduced carbon uptake of a boreal forest ecosystem in Northeast China: Evidence from an 11-year eddy covariance observation

**DOI:** 10.3389/fpls.2023.1119670

**Published:** 2023-01-25

**Authors:** Yujie Yan, Li Zhou, Guangsheng Zhou, Yu Wang, Jiaxin Song, Sen Zhang, Mengzi Zhou

**Affiliations:** ^1^ School of Geo-Science and Technology, Zhengzhou University, Zhengzhou, China; ^2^ State Key Laboratory of Severe Weather, Chinese Academy of Meteorological Sciences, Beijing, China; ^3^ Joint Laboratory of Eco-Meteorology, Chinese Academy of Meteorological Sciences, Zhengzhou University, Zhengzhou, China; ^4^ College of Horticulture and Plant Protection, Henan University of Science and Technology, Luoyang, China

**Keywords:** net ecosystem CO_2_ exchange, eddy covariance, boreal forest, carbon budget, environmental controls, extreme temperature events

## Abstract

Boreal forests, the second continental biome on Earth, are known for their massive carbon storage capacity and important role in the global carbon cycle. Comprehending the temporal dynamics and controlling factors of net ecosystem CO_2_ exchange (NEE) is critical for predicting how the carbon exchange in boreal forests will change in response to climate change. Therefore, based on long-term eddy covariance observations from 2008 to 2018, we evaluated the diurnal, seasonal, and interannual variations in the boreal forest ecosystem NEE in Northeast China and explored its environmental regulation. It was found that the boreal forest was a minor CO_2_ sink with an annual average NEE of -64.01 (± 24.23) g CO_2_ m^-2^ yr^-1^. The diurnal variation in the NEE of boreal forest during the growing season was considerably larger than that during the non-growing season, and carbon uptake peaked between 8:30 and 9:30 in the morning. The seasonal variation in NEE demonstrated a “U” shaped curve, and the carbon uptake peaked in July. On a half-hourly scale, photosynthetically active radiation and vapor pressure deficit had larger impacts on daytime NEE during the growing season. However, temperature had major control on NEE during the growing season at night and during the non-growing season. On a daily scale, temperature was the dominant factor controlling seasonal variation in NEE. Occurrence of extreme temperature days, especially extreme temperature events, would reduce boreal forest carbon uptake; interannual variation in NEE was substantially associated with the maximum CO_2_ uptake rate during the growing season. This study deepens our understanding of environmental controls on NEE at multiple timescales and provides a data basis for evaluating the global carbon budget.

## 1 Introduction

Global climate change, characterized by global warming, is one of the most important issues people face today, and it is essential to significantly reduce greenhouse gas emissions to keep world temperatures 1.5°C above pre-industrial levels (Bosch et al., 2017; Tian et al., 2020; IPCC, 2021). Terrestrial ecosystems have a net carbon uptake of 3.4 ± 0.9 Pg C yr^−1^ from the atmosphere, playing a significant role in the global carbon cycle and reducing global warming (IPCC, 2021). Therefore, it is vital to comprehend the terrestrial ecosystems carbon budget and environmental regulations.

As one of the major terrestrial ecosystems, forests contribute nearly half of the terrestrial production and can effectively reduce CO_2_ accumulation in the atmosphere (Reichstein and Carvalhais, 2019). Among them, boreal forest ecosystems are the second continental biome on Earth and contain approximately 30% of all forests in terms of carbon storage (Pan et al., 2011). Boreal forests have the potential to reduce atmospheric carbon emissions by increasing carbon storage in plants, soil, and wood products and by displacing fossil fuels (Baul et al., 2017; Cintas et al., 2017). Additionally, there is evidence that the boreal forest region is one of the most rapidly warming regions globally (Walsh, 2014). Boreal forests are considered to be more vulnerable to climate change than other ecosystems in the world and are one of the critical climate tipping points of the world, where climate change may cause widespread boreal forest dieback (Bonan, 2008; Lenton et al., 2008; Gauthier et al., 2015; Venalainen et al., 2020; Mckay et al., 2022). Therefore, boreal forests are a key area for global carbon cycle research, and they have attracted increasing attention from researchers in recent years (Venier et al., 2018; Holmberg et al., 2019; Piznak and Backor, 2019; Frelich et al., 2021), but there are still debates on their carbon source/sink issues (Heijmans et al., 2004; Soloway et al., 2017). Boreal forests may function as a net CO_2_ source or sink at the ecosystem scale or may switch between the two states. In a study of an artificial boreal forest in Maoer Mountain, China, the forest absorbed an annual average of 575.66 g CO_2_ from 2008 to 2018 (Liu et al., 2021). The annual average uptake of afforested temperate white pine forest in Ontario was 382.74 g CO_2_ from 2003 to 2013 (Chan et al., 2018). It experienced a transformation from carbon sink to carbon source in the Manitoba boreal black spruce forest, with an annual average release of 66 g CO_2_ (Dunn et al., 2007). In central Sweden, the major boreal forest ecosystem was a constant CO_2_ source, shedding approximately 250.5 g CO_2_ m^-2^ yr^-1^ over a ten-year period (Hadden and Grelle, 2017). In summary, boreal forests demonstrate large spatial variations from the net ecosystem exchange perspective. Since the establishment of ChinaFlux in 2002, carbon flux observations have been conducted in diverse forests (Yu et al., 2006; Zhu et al., 2006; Yu et al., 2014; Wang et al., 2016), but there have been few reports on the original boreal forest ecosystem, except for the Genhe station in Inner Mongolia (Li and Zhang, 2015). Research on carbon flux dynamics in boreal forest ecosystems in China is still in its infancy.

Some studies have demonstrated that the factors controlling net ecosystem CO_2_ exchange (NEE) on different scales are quite different (Fu et al., 2017b, Wu JK et al., 2020). On half-hourly and daily scales, radiation and temperature are often considered the dominant factors in NEE variation, but precipitation and soil moisture have stronger effects on NEE in some arid regions (Park et al., 2011; Li et al., 2014; Rebane et al., 2019, Wang Y et al., 2019). NEE interannual variation (IAV) is predominantly influenced by physiological (e.g., maximum carbon uptake rate) and phenological factors (e.g., length of the growing season); however, some studies have indicated that it is strongly associated with annual mean temperature and cumulative precipitation (Oquist et al., 2014; Xu et al., 2019; Jia et al., 2021; Liu et al., 2021). Although, owing to the lack of long-term continuous high-quality observational data, there are few systematic studies on the factors influencing NEE at diverse scales in boreal forest ecosystems, and the control mechanism is still obscure (Venalainen et al., 2020).

Additionally, the boreal forest ecosystem is situated in the middle and high latitudes of the Northern Hemisphere, where rapid climate change and frequent extreme temperatures are common (Seneviratne et al., 2021). Temperature has a strong influence on plant photosynthesis, soil microbial activity, organic matter decomposition, phenology, and consequently, NEE (Horemans et al., 2020; Kang et al., 2020). When extremely high temperatures occurred in Southern Australia during the summer of 2013, the maximum daily carbon uptake of temperate forests in the region decreased, which in turn affected the cumulative NEE during the growing season (Van Gorsel et al., 2013). Net carbon uptake during the growing season decreased in 2003 due to the high temperatures in European forests. For example, carbon uptake in the growing season decreased by approximately 160 g C m^-2^ yr^-1^ in the evergreen coniferous forest of Italy (Ciais et al., 2005). In the summer of the same year, the subtropical region of East Asia also experienced extremely high temperatures, which significantly reduced forest ecosystems carbon uptake during the growing season (Saigusa et al., 2010; Wen et al., 2010). Previous discussions of the impact of extreme temperatures on NEE have majorly focused on the tropics and subtropics, whereas boreal forests have been ignored (Yuri et al., 2021). Considering that the scale and intensity of extreme temperature events may increase in the future, to accurately evaluate the carbon sink capacity of forest ecosystems, it is essential to elucidate the response characteristics of net carbon uptake of boreal forests to extreme temperatures (Meehl and Tebaldi, 2004; Shao et al., 2022).

Therefore, based on 11-year eddy covariance observations from a boreal forest ecosystem in China, this study aimed to (1) quantify the dynamic characteristics of diurnal, seasonal, and interannual variations of CO_2_ fluxes in the boreal forest; (2) comprehend the environmental regulations on CO_2_ flux changes at diverse time scales; and (3) explore the effects of extreme temperature on the boreal forest NEE.

## 2 Materials and methods

### 2.1 Site description

The research was conducted at the Research Station for Ecosystem Positioning of Chinese Northern Coniferous Forests (51°46’52”N, 126°01’04”E; 773 m a.s.l.), maintained by the Institute of Botany, the Chinese Academy of Sciences, and the Heilongjiang Huzhong National Nature Reserve Administration. The area had cold, dry winters and hot, rainy summers, with a typical continental monsoon climate. The long-term annual average air temperature (1991–2020) was -2.95°C, and the annual average precipitation was approximately 500.8 mm. Additionally, there were significant seasonal (approximately 70% of precipitation occurring in the summer) and interannual (272.4–748.8 mm) variations in precipitation. *Larix gmelinii* was the only dominant species at the research site, while *Betula platyphylla* was the primary companion tree species. *Ledum palustre*, *Vaccinium vitis-idaea*, and *Rhododendron dauricum* constituted most of the understory.

### 2.2 Eddy-covariance and supporting measurements

Using an Open Path Eddy Covariance (OPEC) system installed at 35.0 m, carbon (the net ecosystem exchange of CO_2_, NEE) and water fluxes were observed. The OPEC system included a 3-D sonic anemometer (CAST3, Campbell Scientific, Inc., Logan, UT, USA), which measures wind speed and virtual temperature, and an infrared gas analyzer (IGRA; LI-7500, LI-COR, Inc., Lincoln, NE, USA), which monitors changes in CO_2_ and water vapor densities. The sampling rate for flux data was 10 Hz.

Additionally, a quantum sensor (LI-190SB, LI-COR, Inc., Lincoln, NE, USA) and a four-component net radiometer (CNR1, Kipp & Zonen, Crop., Delft, Holland) were utilized to measure photosynthetically active radiation (PAR) and net radiation (Rn) 32 m above the ground. A thermo-hygrometer was used to measure the relative humidity (RH) and air temperature (T_a_) (HMP45C, Vaisala, Inc., Helsinki, Finland). Soil temperature (T_s_; 107 L, Campbell Scientific, Inc., Edmonton, Alberta, Canada) at six depths (0, 0.05, 0.1, 0.15, 0.2, and 0.3 m) and volumetric soil water content (SWC, CS616, Campbell Scientific, Inc., UT, USA) at 0.08 m were measured. A rain gauge (52203, RM Young, Inc., Traverse City, MI, USA) mounted on the top of the tower was utilized to measure precipitation (37 m). All meteorological data were collected as 30 min averages using a data logger (CR23XTD, Campbell Scientific, Inc., UT, USA).

### 2.3 Post-processing of eddy-covariance measurements

Half-hourly CO_2_ fluxes were computed and corrected from raw OPEC recordings using EddyPro 7.0.6 (LI-COR Inc., Lincoln, NE). The major post-processing procedures included spike removal, double-coordinate rotation, corrections for frequency losses and sonic temperature, corrections for density fluctuations (i.e., the Webb–Pearman–Leuning correction), and flux computation (Webb et al., 1980). It should be noted that because the infrared gas analyzer (LI-7500) surface temperature differs from the air temperature due to electronic instrument heating, solar radiation, and radiative cooling, the Burba Equation should be utilized for further correction during processing (Burba et al., 2008). Rain events, outliers (Papale et al., 2006), and nighttime data that occurred under low friction velocity (u*) circumstances were used to filter the flux data during these times. The u* threshold was estimated using the method described by Zhu et al. (2006). Using the marginal distribution sampling (MDS) technique, flux data gaps were filled (Reichstein et al., 2005). A positive (negative) NEE value indicates that the ecosystem is a carbon source (sink). The growing season usually started in early May and ended in late September; the remaining period was defined as the non-growing season.

### 2.4 Data analysis

The maximum CO_2_ uptake (MCU) and release rate (MCR) obtained from the smoothed daily NEE curves were considered as ecosystem physiological metrics (Fu et al., 2019). A Savitzky–Golay filter was applied to smoothen the daily NEE (Savitzky and Golay, 1964). The number of days with a net carbon uptake (NEE < 0 g CO_2_ m^-2^ d^-1^) was defined as the net CO_2_ uptake period (CUP).

According to the 27 extreme climate indices defined by the Expert Team on Climate Change Detection and Indices (ETCCDI) (Peterson and Manton, 2008), the relative threshold index computed from the maximum/minimum temperature in the boreal forest ecosystem growing season from 1991 to 2020 was 30°C/-4°C. Temperature extremes occur when daily maximum/minimum temperatures during the growing season are above the high threshold (30°C) or below the low thresholds (-4°C). The number of days when it occurred was defined as the number of high/low-temperature days, and the period when the number of high/low-temperature days lasted for six days or more was defined as an extreme high/low-temperature event. The effect of extreme temperatures on NEE during the growing season was characterized by the relative rate of change (***α***) of NEE (Tatarinov et al., 2016).

## 3 Result

### 3.1 Environmental conditions

#### 3.1.1 Heat-related environmental factors

**Figure 1:**
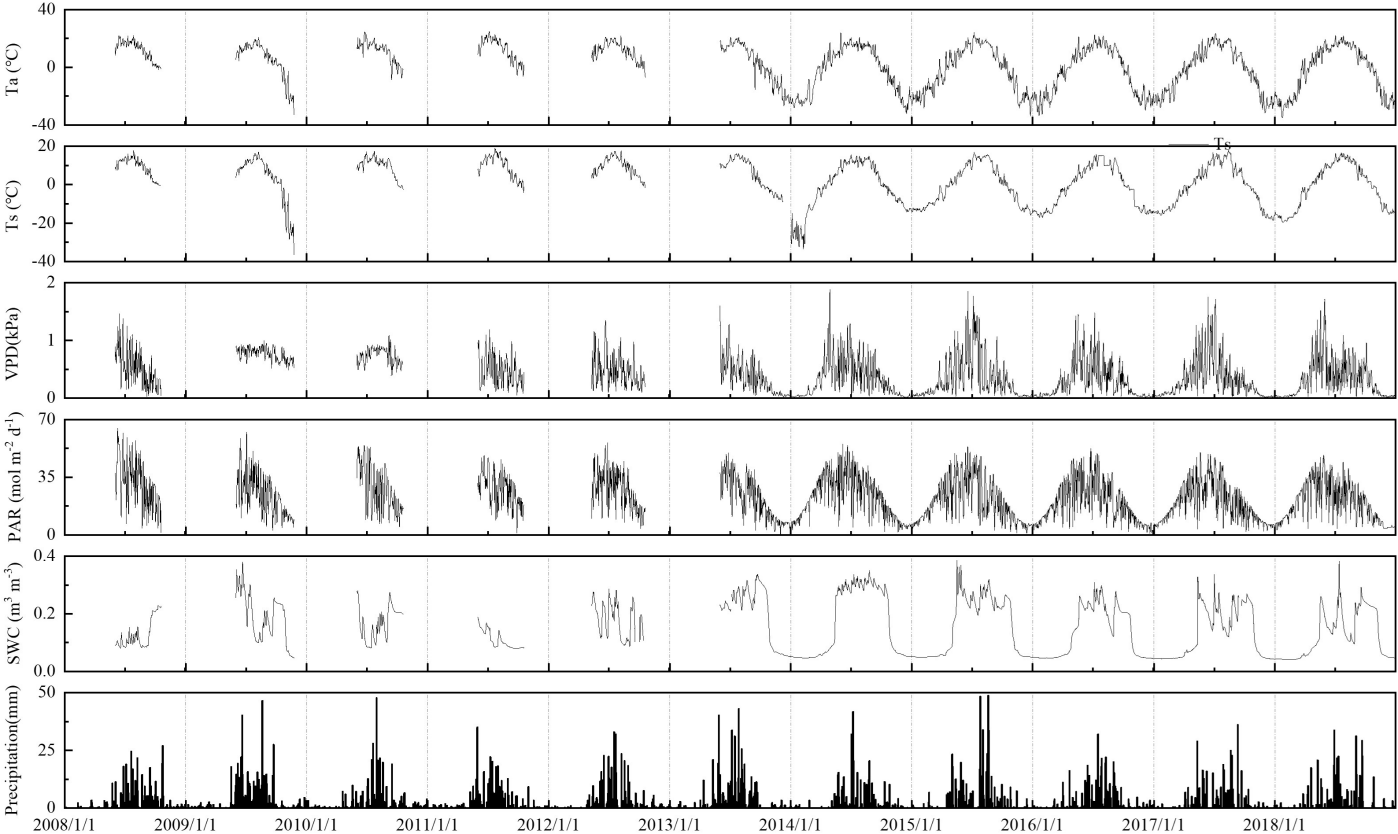
Dynamic changes in environmental factors in the boreal forest ecosystem. PAR, photosynthetically active radiation, VPD, vapor pressure deficit, T_a_, air temperature, T_s_, soil temperature, SWC, volumetric soil water content, PPT, precipitation.

T_a_, T_s_, and PAR values of the boreal forest ecosystem demonstrated clear seasonal variations (**Figure 1**). The daily average T_a_ ranged from -34.02 to 24.11°C, and its seasonal variation was similar among different years. The daily average T_s_ ranged from -19.88 to 17.84°C, and its overall T_s_ trend was consistent with that of T_a_, but the seasonal amplitude of T_s_ was smaller than T_a_. The average T_s_ was higher than T_a_, and the seasonal variation in T_s_ was smoother, exhibiting hysteresis. T_a_ and T_s_ at the end of November 2009 and January-February 2014 were lower than those in other years. The daily accumulative PAR ranged from 0.72 to 55.94 mol m^-2^ d^-1^, with a peak appearing from May to July. PAR in the growing seasons of 2011 and 2018 was usually small, which was related to excessive rainfall during the growing seasons in these years.

#### 3.1.2 Moisture-related environmental factors

The vapor pressure deficit (VPD) ranged from 0.13 to 1.88 kPa, and the maximum VPD value appeared from June to August. Due to heavy rains during the growing season in 2009, 2010, and 2011, the VPD was lower than 1.0 kPa. Precipitation varied greatly over the years, with more precipitation in summer, which accounted for more than 50% of the annual precipitation. The variation in the daily average SWC was closely related to precipitation and ranged from 4.1 to 34.1%. SWC in the entire growing season in 2014 remained above 25%, which was related to the greater number of rainfall days in the growing season of this year.

### 3.2 Diurnal variations in NEE and its environmental regulation

**Figure 2:**
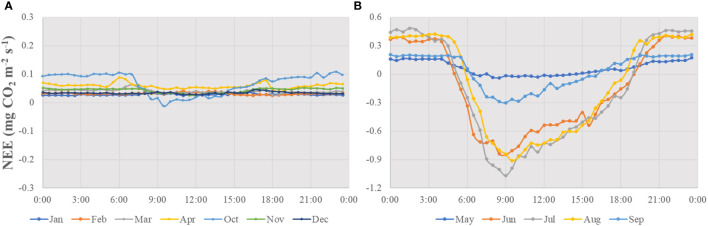
Diurnal variations in NEE (net ecosystem CO_2_ exchange) on a monthly average from 2008 to 2018: **(A)** non-growing season, **(B)** growing season. Jan, January, Feb, February, Mar, March, Apr, April, Jun, June, Jul, July, Aug, August, Sep, September, Oct, October, Nov, November, Dec, December.

According to the monthly average diurnal variations in NEE, the diurnal amplitude of NEE during the growing season was noticeably higher than that in the non-growing season (**Figure 2**). NEE during the non-growing season demonstrated no clear diurnal variation (**Figure 2A**), and the boreal forest acted as a net carbon source. However, a significant diurnal pattern resembling a “U” curve (**Figure 2B**) was observed during the growing season, with positive values (net carbon release) at night and negative values (net carbon uptake) in the daytime. The peak of carbon uptake appeared at 8:30–9:00 in the morning, ranged from -0.03 (May) to -0.91 mg CO_2_ m^-2^ s^-1^ (August). The earliest time for the NEE value to switch from positive to negative was in June and July (5:30), and the longest daily carbon sequestration time (14 h) occurred in June.

According to the determinative coefficient, PAR and VPD were the prominent environmental control factors, and the order of intensity of influence of each environmental factor on daytime NEE in the growing season was PAR > VPD >T_a_ >T_s_ >SWC (**Table 1**). The direct and indirect path coefficients of the environmental factors reflect whether the factor affects NEE majorly through direct or indirect ways. From the path coefficient value, PAR and T_a_ have a direct impact on NEE. The PAR direct path coefficients and T_a_ were -0.354 and -0.220, respectively, which were larger than their indirect path coefficients (0.016, 0.00), indicating that these two driving factors had significant effects during the daytime during the growing season. However, SWC had an indirect impact, which affected daytime NEE during the growing season by influencing other factors, but the determinative coefficient was small and the influence was not significant. There was little difference between the direct and indirect impacts of VPD and T_s_. The direct and indirect path coefficients of VPD were 0.392 and -0.344, respectively, which inhibited CO_2_ uptake. T_s_ had equivalent direct and indirect effects on NEE and their combined effects contributed to CO_2_ uptake.

**Table 1:** Direct and indirect path coefficients of environmental factors on NEE (net ecosystem CO_2_ exchange) diurnal variation.

Period	Factors	Related coefficient	Direct path coefficient	The sum of indirect path coefficient	Determinative coefficient
Daytime in growing season	PAR	-0.354	-0.369	0.015	0.1251
	VPD	0.048	0.392	-0.344	0.1160
	T_a_	-0.220	-0.220	0.000	0.0484
	T_s_	-0.225	-0.125	-0.100	0.0406
	SWC	0.074	0.001	0.073	0.0002
Nighttime in growing season	VPD	-0.025	-0.080	0.055	0.0024
	T_a_	0.212	-0.147	0.359	0.0839
	T_s_	0.267	0.443	-0.176	0.0403
	SWC	0.094	0.124	-0.030	0.0079
Non-growing season	VPD	0.184	0.187	-0.003	0.0338
	T_a_	0.188	-0.252	0.440	0.1582
	T_s_	0.254	0.343	-0.089	0.0566
	SWC	0.136	0.029	0.107	0.0071

PAR, photosynthetically active radiation, VPD, vapor pressure deficit, T_a_, air temperature, T_s_, soil temperature, SWC, volumetric soil water content.

Temperature had the greatest impact on NEE during the growing season at night and during the non-growing season (**Table 1**). In general, ecosystem respiration is more active and releases more CO_2_ at high temperatures. According to the determinative coefficient, the effect of T_a_ on NEE was majorly indirect, by affecting T_s_ and VPD, whereas that of T_s_ was direct. Additionally, during the non-growing season, NEE was majorly affected by T_a_ and was relatively less affected by T_s_.

### 3.3 Seasonal variations in NEE and its environmental regulation

**Figure 3:**
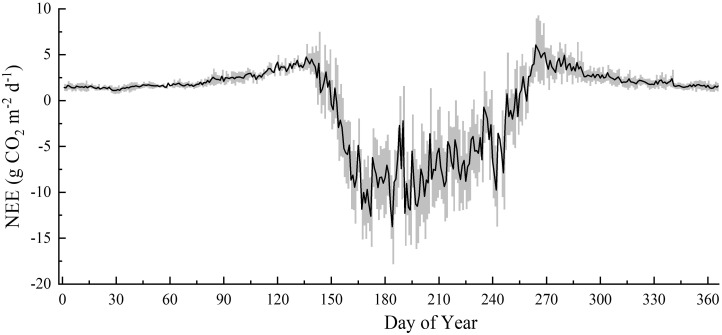
Average daily NEE (net ecosystem CO_2_ exchange) seasonal variation from 2014 to 2018.

The boreal forest station initially only observed fluxes in the growing season, and carbon fluxes have been observed throughout the year since 2014. The seasonal variations in NEE and its environmental regulation were evaluated based on data from 2014 to 2018. The seasonal variation in NEE in boreal forests demonstrated a unimodal curve (**Figure 3**), which indicated it to be a CO_2_ source during the non-growing season and a CO_2_ sink during the growing season. Boreal forests began to germinate in early May, and as the leaves grew, photosynthesis and respiration were both enhanced, but the respiration rate was higher than that of photosynthesis, resulting in a small CO_2_ emission peak (5.69 ± 1.75 g CO_2_ m^-2^ d^-1^) in late May. The CO_2_ uptake in boreal forests usually peaked in early July (-20.35 ± 2.91 g CO_2_ m^-2^ d^-1^). The growth of the boreal forests decelerated at the end of August, with the net carbon uptake gradually decreasing. In mid-September, boreal forests switched from carbon sinks to sources and reached their peak CO_2_ emissions (7.58 ± 2.08 g CO_2_ m^-2^ d^-1^) in late September. The plants entered dormancy from October to April of the following year and the metabolic activity of the ecosystem was minimal. During the non-growing season, there was no discernible variation in CO_2_ flux, with the NEE varying from 0 to 3.5 g CO_2_ m^-2^ d^-1^.

During the growing season, T_a_ was the dominant controlling factor for seasonal variation in NEE in boreal forests, followed by PAR. On a daily scale, the boreal forests carbon uptake increased with increasing T_a_ and PAR (**Table 2**). T_a_ exerted a direct effect on the seasonal variation in NEE, whereas PAR had a greater indirect effect. During the non-growing season, the daily NEE was predominantly influenced by T_s_ and SWC, both of which positively encouraged CO_2_ release, exerting direct effects on the NEE.

**Table 2:** Direct and indirect path coefficients of environmental factors on NEE (net ecosystem CO_2_ exchange) seasonal variation.

Period	Factors	Related coefficient	Direct path coefficient	The sum of indirect path coefficient	Determinative coefficient
Growing season	PAR	-0.220	-0.080	-0.140	0.0274
	T_a_	-0.551	-0.530	-0.021	0.3029
	SWC	-0.119	-0.060	-0.059	0.0002
Non-growing season	SWC	0.633	0.350	0.283	0.3206
	T_s_	0.690	0.490	0.200	0.4361

PAR, photosynthetically active radiation, T_a_, air temperature, T_s_, soil temperature, SWC, volumetric soil water content.

### 3.4 Interannual variations in NEE and its environmental regulation

**Figure 4:**
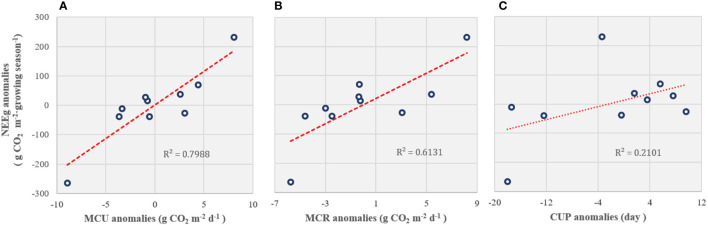
The relationship between the carbon flux (NEE_g_) anomalies during the growing season and **(A)** the maximum CO_2_ uptake rate (MCU), **(B)** maximum CO_2_ release rate (MCR), and **(C)** carbon uptake period (CUP) anomalies.

On an annual scale, boreal forest ecosystems are carbon sinks for atmospheric CO_2_ (**Table 3**). The mean NEE accumulation in the growing season from 2008 to 2018 (2013 was excluded) was -676.01 (± 134.07) g CO_2_ m^-2^ growing season^-1^, and mean annual carbon budget from 2014 to 2018 was -64.01 (± 24.23) g CO_2_ m^-2^ yr^-1^. There were no significant relationships between the environmental factors and annual cumulative NEE. However, the yearly NEE anomalies during the growing season (NEE_g_) were significantly correlated with the maximum CO_2_ uptake rate (MCU) (*p* < 0.001; **Figure 4A**) and maximum CO_2_ release rate (MCR) anomalies (*p* < 0.01; **Figure 4B**) but not with the carbon uptake period (CUP) anomalies (*p* = 0.182; **Figure 4C**).

**Table 3:** Interannual variation in cumulative NEE (net ecosystem CO_2_ exchange) during the growing season and throughout the year.

Year	Accumulated NEE in the growing season (g CO_2_ m^-2^ growing season^-1^)	Accumulated NEE throughout the year (g CO_2_ m^-2^ yr^-1^)
2008	-983.841	\
2009	-440.372	\
2010	-710.226	\
2011	-682.285	\
2012	-656.673	\
2014	-697.633	-28.492
2015	-634.698	-71.689
2016	-601.431	-72.245
2017	-643.571	-54.232
2018	-709.366	-93.410

### 3.5 Impact of extreme temperature events on NEE

The boreal site experienced extremely high temperatures for 5–24 d during each growing season from 2014 to 2018 (**Table 4**). The cumulative growing-season NEE was found to decrease only in 2014, 2015, and 2017 when the number of extreme high-temperature days lasted long enough to form an extreme heat event. Conversely, the cumulative growing-season NEE did not decrease in 2016 and 2018, when the number of consecutive days of extremely high temperatures was insufficient to form an extreme heat event. After a short period of extreme high temperatures, the NEE recovered quickly and the cumulative NEE in the growing season increased. In comparison, the effect of extremely low temperatures on NEE was different. The occurrence of extreme low-temperature days lead to a decrease in the cumulative growing-season NEE even if it did not last long enough to constitute an extreme low-temperature event. The decreasing percentage of NEE increased with the number of extreme low-temperature days (**Table 4**).

**Table 4:** Extreme temperature day/event and their effects on cumulative NEE (net ecosystem CO_2_ exchange) during the growing season.

Year	Type	Number of extreme temperature days	Number of extreme temperature events	Duration	*α*
2014	Heat	15	1	6.27–7.02	-1.45%
	Cold	6	0	\	-2.26%
2015	Heat	24	1	7.05–7.20	-5.94%
	Cold	7	0	\	-1.29%
2016	Heat	5	0	\	1.56%
	Cold	15	1	8.25–8.30	-5.76%
2017	Heat	17	2	6.24–6.26	-6.50%
	Cold	12	0	7.02–7.08	-4.22%
2018	Heat	9	0	\	1.71%
	Cold	3	0	\	1.51%

α, relative rate of change of NEE (net ecosystem CO_2_ exchange) during the growing s.

## 4 Discussion

### 4.1 Magnitude of NEE

Owing to variations in ecosystem types, climatic conditions, and subsurface materials, NEE diurnal variation typically exhibits distinct patterns and magnitudes (Zhou et al., 2009, Wu JK et al., 2020, Zhang et al., 2020). In this study, the diurnal NEE dynamics in the boreal forest ecosystem displayed a single peak curve, and the peak CO_2_ uptake period occurred between 8:30 and 9:30 in the morning. This is consistent with previous studies of Korean pine and boreal-leaved mixed forests in the Changbai Mountains (Guan et al., 2006). The maximum carbon uptake rate at this research site during the growing season attained -1.068 mg CO_2_ m^-2^ s^-1^, which is substantially larger than those reported for a *Larix gmelinii* forest in Central Siberia (-0.475 mg CO_2_ m^-2^ s^-1^, Nakai et al., 2008), and for a hemiboreal forest ecosystem in Estonia (-0.792 mg CO_2_ m^-2^ s^-1^, Rebane et al., 2020), but is similar to that from a *Larix gmelinii* forest in Daxing’anling Mountains (-1.09 mg CO_2_ m^-2^ s^-1^, Li and Zhang, 2015).

In our study, the seasonal variation in NEE was a U-shaped curve with a peak from June to July, which is consistent with earlier studies (Welp et al., 2007; Gill et al., 2015; Frelich et al., 2021). However, higher temperatures (17.54°C in August 2015 and 17.33°C in August 2017 compared to the 30-year average value of 15.38°C from 1991 to 2020) and ample precipitation (221.4 mm in August 2015 and 155.6 mm in August 2017 compared to the 30-year average value of 115.1 mm from 1991 to 2020) led to the occurrence of an additional small peak in August of 2015 and 2017. In the growing season from 2008 to 2018 (2013 was excluded), the average daily NEE accumulation was -4.69 (± 13.07) g CO_2_ m^-2^ d^-1^, which was within the range reported in previous studies (**Table 5**).

**Table 5:** Comparison of growing season and annual CO_2_ budget among diverse forest ecosystems.

Site	Ecosystem type	Latitude	Daily average of NEE in growing season (g CO_2_ m^-2^ d^-1^)	NEE in the whole year (g CO_2_ m^-2^ yr^-1^)	Period	Source
Ontario, Canada	Afforested temperate white pine forest	42°39’	\	-382.74	2003– 2016	(Chan et al., 2018)
Manitoba, Canada	Boreal black spruce forest	55°53’	\	-212–308	1995– 2004	(Dunn et al., 2007)
Inner Mongolia, China	Cold temperate coniferous forest	50°49’	-2.42	\	2013	(Li and Zhang, 2015)
Jilin, China	Broad-leaved Korean pine mixed forest	42°24’	-7.05	-823.16	2003–2004	(Zhang et al., 2006a)
Harbin, China	Temperate deciduous broadleaved forest	45°24’	\	-575.66	2008–2018	(Liu et al., 2021)
Beijing, China	Deciduous broad-leaved forest	40°30’	\	-407.00	2019	(Li et al., 2019)
Heilongjiang, China	Cold temperate coniferous forest	51°46’	-4.69	-64.01	2008–2018	This study

The boreal forest had an annual CO_2_ budget of -64.01 ± 24.23 g CO_2_ m^-2^ yr^-1^ over the studied years, and it acted as a CO_2_ sink. Previous studies on the forest ecosystems annual CO_2_ budget had a variety of magnitude values, most of which were carbon sinks (ranging from -382.74 g to -823.16 g CO_2_ m^-2^ yr^-1^). A few of them shifted from a carbon source to a sink, such as the boreal black spruce forest in Manitoba, Canada (**Table 5**). Comparatively, the boreal forest in this study was a relatively small carbon sink, mostly due to the colder climate and shorter growing season at higher latitudes.

### 4.2 Environmental regulations of NEE variations

#### 4.2.1 Effects of radiation on NEE

**Figure 5:**
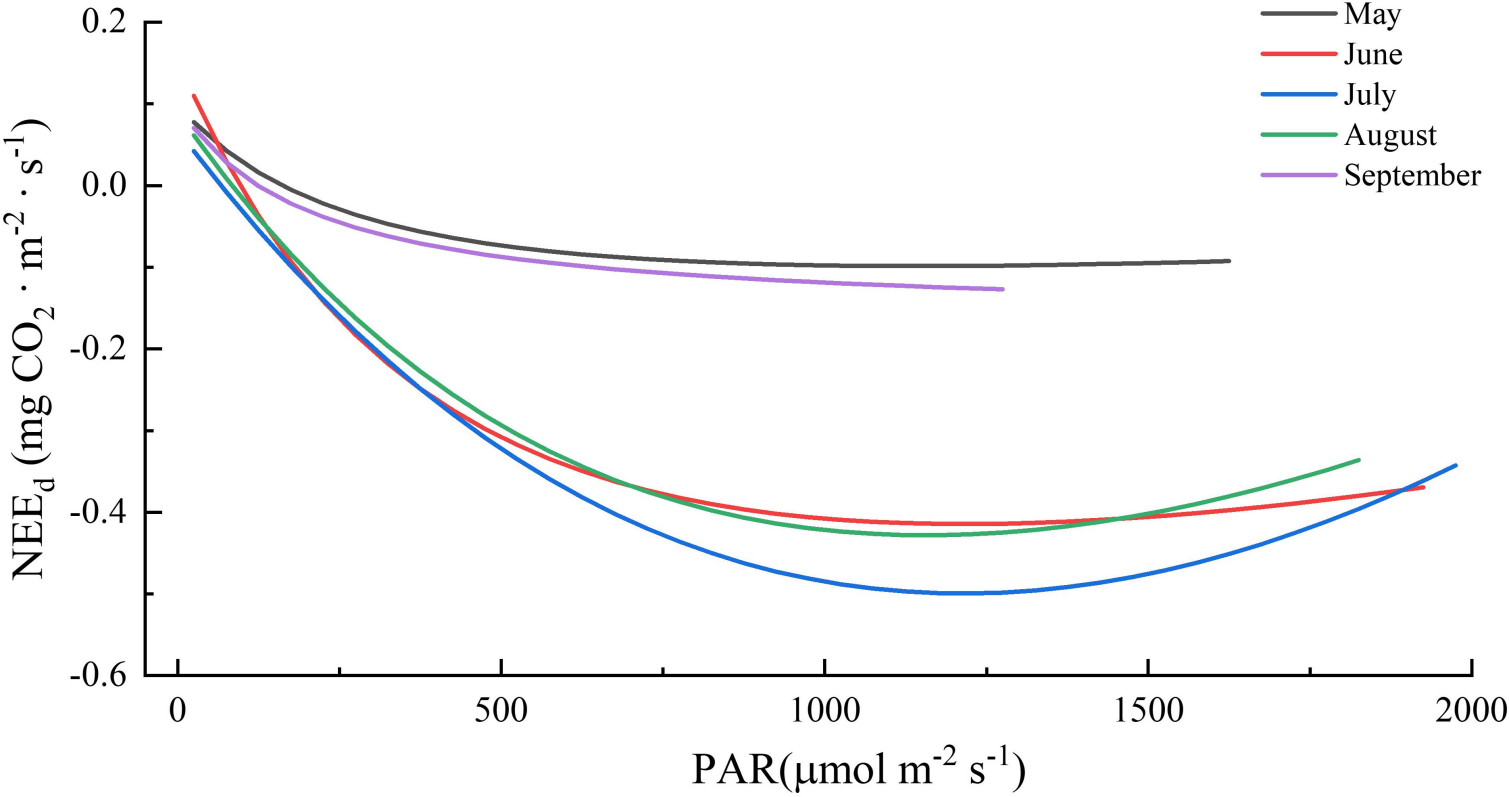
Responses of NEE_d_ (net ecosystem CO_2_ exchange in the daytime) to photosynthetically active radiation (PAR) during the growing seasons in boreal forest ecosystem.

Radiation, particularly PAR, has a significant impact on ecosystem photosynthesis and consequently, NEE (Baldocchi, 2014; Wang Y et al., 2019). This study also demonstrated that PAR elucidated most of the variance in half-hourly daytime NEE during the growing season (**Table 1**) and that the Michaelis–Menten model was a good fit to the relationship between daytime NEE and PAR (*r* ^2^ > 0.76, *p* < 0.01; details in **Table 6** and **Figure 5**). The parameters estimated from the model, including the initial light utilization rate (***α***), maximum photosynthetic rate (***A_max_***), and daytime respiration intensity (***R_d_***), are usually used as plant photosynthetic capacity indicators. In this study, the *α* values ranged from 0.0009 to 0.0019 mg CO_2_ μmol photon^-1^, with its maximum value (0.0019 mg CO_2_ μmol photon^-1^) appearing in June; ***A_max_*** ranged from 0.0985 to 0.4995 mg CO_2_ m^-2^ s^-1^, with its maximum value appearing in July (0.4995 mg CO_2_ m^-2^ s^-1^), *R_d_* ranged from 0.0994 to 0.1555 mg CO_2_ m^-2^ s^-1^, with its maximum value appearing in June (0.1555 mg CO_2_ m^-2^ s^-1^). Therefore, ecosystem carbon models should take into account the seasonal dynamics of photosynthetic capacity parameters to accurately estimate the carbon budget. Among them, ***A_max_*** represents photosynthesis intensity in the ecosystem under saturated light intensity and reflects the impact of biochemical processes and physiological conditions on photosynthesis in the ecosystem (Zhou et al., 2017). ***A_max_*** of boreal forests in this study was lower than that in previous studies, which could account for the comparatively low yearly carbon uptake of boreal forests (Li and Zhang, 2015; Li et al., 2019; Wang Q et al., 2019).

**Table 6:** Parameters of NEE (net ecosystem CO_2_ exchange) light response curves during the growing seasons in the boreal forest ecosystem.

Month	*α*	*A_max_*	*R_d_*	*R^2^*	*p*
	(mg CO_2_ ·μmol photon^-1^)	(mg CO_2_ · m^-2^ · s^-1^)	(mg CO_2_ · m^-2^ · s^-1^)		
5	0.0009	0.0985	0.0994	0.7625	<0.01
6	0.0019	0.4144	0.1555	0.9337	<0.01
7	0.0011	0.4995	0.0681	0.9225	<0.01
8	0.0011	0.4282	0.0894	0.9391	<0.01
9	0.0013	0.1621	0.0991	0.8154	<0.01

α, initial light utilization rate, A_max_, maximum photosynthetic rate, R_d_, daytime respiration intensity.

#### 4.2.2 Effects of temperature on NEE

Temperature was the most important controlling factor for night NEE in the growing season and for the NEE in the non-growing season (**Table 1**). We determined the model parameters for each month using the Lloyd and Taylor model for simulating the response of ecosystem respiration to varying soil temperatures (**Table 7**). Respiration at the reference temperature (***R_Tref_***) gradually increased as the temperature rose, peaking in July; except for May, wherein in the initial growth stage, the temperature sensitivity (***Q_10_***) continuously decreased as the temperature increased (Chen et al., 2010; Han and Jin, 2018). As a result, ***Q_10_*** was higher during the non-growing season than during the growing season. The rapid increase in the root and rhizosphere respiration rates at the beginning of the growing season might be the cause of the increased ***Q_10_*** in May (Shabaga et al., 2015).

**Table 7:** Monthly respiration-temperature response equation parameters of boreal forest ecosystems.

Month	*R_Tref_*	*T_0_*	*Q_10_*	*R^2^*
1	0.0268	146.2057	1.241	0.45
2	0.0374	166.0404	1.243	0.41
3	0.0337	138.7903	1.205	0.49
4	0.0300	135.5546	1.176	0.47
5	0.1066	189.0448	1.441	0.80
6	0.1999	129.2273	1.131	0.55
7	0.2443	157.8814	1.199	0.39
8	0.2357	184.8783	1.152	0.72
9	0.1232	113.9823	1.141	0.47
10	0.0829	159.9415	1.267	0.63
11	0.0310	143.6633	1.278	0.38
12	0.0290	164.0277	1.356	0.36

R_Tref_, the respiration at the reference temperature, T_0_, the test constant, Q_10_, the temperature sensitivity.

#### 4.2.3 Effects of extreme temperature on NEE

Extreme temperatures have diverse effects on ecosystem photosynthesis and respiration; the net carbon uptake in the growing season is reduced when the adverse impact on photosynthesis is greater than that on respiration (Tatarinov et al., 2016; Krasnova et al., 2022). For example, in the Qianyanzhou subtropical coniferous forest, an extreme heat event lasting 36 days resulted in a 6.7% decrease in the annual ecosystem carbon uptake (Zhang et al., 2018). In a mixed conifer-broadleaved forest in Southern Estonia, an extreme high-temperature event that lasted for 19 days in 2018 caused the forest to change from a carbon sink to a carbon source (Krasnova et al., 2022). In this study, we found that the impact of extreme temperatures on carbon uptake was related to its duration and intensity. When extreme temperature events with short durations occur, if the temperature and water conditions in the early and late stages are suitable, the forest will maintain better resilience, and the net carbon uptake will reach the original level (Ciais et al., 2005; Zhang et al., 2018). For example, in the studied boreal forest, the cumulative growing season NEE did not decrease in 2016 and 2018 when the number of consecutive days of extreme high temperatures was insufficient to form an extreme heat event. Meanwhile, given that extreme temperature events often occur together with extreme drought, it is essential to evaluate the changes in carbon uptake under the synergy of the two (Wu HD et al., 2020, Yan et al., 2020).

## 5 Conclusion

The boreal forest ecosystem served as a weak carbon sink with an annual average NEE accumulation (2014–2018) of -64.01 ( ± 24.23) g CO_2_ m^-2^ yr^-1^ and growing season average (2008–2018; 2013 was excluded) of -676.01 (± 134.07) g CO_2_ m^-2^ growing season^-1^. Additionally, diurnal, seasonal, and interannual variations in NEE had obvious dynamic characteristics. In the growing season, PAR was the primary controlling factor of daytime NEE on a half-hourly scale, whereas T_a_ dominated the seasonal NEE variation on a daily scale. Conversely, T_s_ consistently had the greatest effect on non-growing season NEE across the half-hourly and daily scales. The interannual variation in NEE in the growing season was significant in relation to the MCU but not to the environmental factors and CUP. Extreme temperature events can reduce boreal forests carbon uptake, and the impact of extreme temperature on carbon uptake during the growing season is related to its duration and intensity.

## Data availability statement

The original contributions presented in the study are included in the article/supplementary material. Further inquiries can be directed to the corresponding authors.

## Author contributions

Conceptualization, LZ and GZ; methodology, YY and LZ; investigation, JS, YY and SZ; data curation, JS MZ and YW; writing—original draft preparation, YY; funding acquisition, LZ and GZ. All authors contributed to the article and approved the submitted version.
